# Advanced assessment of the physicochemical characteristics of Remicade® and Inflectra® by sensitive LC/MS techniques

**DOI:** 10.1080/19420862.2016.1193661

**Published:** 2016-06-03

**Authors:** Jing Fang, Catalin Doneanu, William R. Alley, Ying Qing Yu, Alain Beck, Weibin Chen

**Affiliations:** a Waters Corporation, Milford, MA, USA; b Centre d'Immunologie Pierre Fabre (CIPF), Saint-Julien-en-Genevois, France

**Keywords:** Host cell proteins, higher order structure, hydrogen deuterium exchange, HDMS^E^, ion mobility spectrometry, N-linked glycans, QTof, *Rapi*Fluor-MS, UPLC, 2D-LC

## Abstract

In this study, we demonstrate the utility of ultra-performance liquid chromatography coupled to mass spectrometry (MS) and ion-mobility spectrometry (IMS) to characterize and compare reference and biosimilar monoclonal antibodies (mAbs) at an advanced level. Specifically, we focus on infliximab and compared the glycan profiles, higher order structures, and their host cell proteins (HCPs) of the reference and biosimilar products, which have the brand names Remicade® and Inflectra®, respectively. Overall, the biosimilar attributes mirrored those of the reference product to a very high degree. The glycan profiling analysis demonstrated a high degree of similarity, especially among the higher abundance glycans. Some differences were observed for the lower abundance glycans. Glycans terminated with N-glycolylneuraminic acid were generally observed to be at higher normalized abundance levels on the biosimilar mAb, while those possessing α-linked galactose pairs were more often expressed at higher levels on the reference molecule. Hydrogen deuterium exchange (HDX) analyses further confirmed the higher-order similarity of the 2 molecules. These results demonstrated only very slight differences between the 2 products, which, interestingly, seemed to be in the area where the N-linked glycans reside. The HCP analysis by a 2D-UPLC IMS-MS approach revealed that the same 2 HCPs were present in both mAb samples. Our ability to perform these types of analyses and acquire insightful data for biosimilarity assessment is based upon our highly sensitive UPLC MS and IMS methods.

## Introduction

Continuing the trend of the previous 3 decades, substantial effort is being invested in the development of monoclonal antibodies (mAbs) as pharmaceutical products. With high specificity, long serum half-lives, and the capability of being produced with high yields and quality, mAbs are increasingly appearing on the drug market.^1^ These large molecules, which have masses of ∼150 kDa, can be designed for specific targets, and several of their physicochemical properties can be predictably controlled.^2^ While very effective, they remain very expensive. However, over the coming years, the patents for several of these biopharmaceuticals are set to expire. This has ignited substantial interest in the development of so-called biosimilar molecules.^3^ These more affordable alternatives, according to the Food and Drug Administration, are “highly similar to the reference product notwithstanding minor differences in clinically inactive components” and have “no clinically meaningful differences between the biological product and the reference product in terms of the safety, purity, and potency of the product.”^4^

The development of biotherapeutic mAbs is a challenging process. MAbs are produced in carefully selected cell lines, and even when generated in the same expression system using the same culture conditions, slight perturbations can lead to distinct product profiles for different batches.^5^ Therapeutic molecules of this size have highly complex secondary and tertiary structures and are often post-translationally modified. Among the key modifications is glycosylation, which is considered a Critical Quality Attribute by the health regulatory authorities.^6^ Glycans are key attributes known to influence a number of physicochemical characteristics, including serum residence time, antibody-dependent cell-mediated cytotoxicity, and certain glycan epitopes (the presence of N-glycolylneuraminic acid and α-linked galactose pairs) may induce an immunogenic response.^7^ Compounding the challenges of producing biopharmaceuticals in cell lines are the process-related impurities that can be present in the final product, albeit at low concentrations, even after extensive purification. Of particular concern are the host cell proteins (HCPs). Those proteins, which can elicit an immunogenic response, are native to the cell line used to produce the mAb, and regulatory guidelines require that they be identified and quantified.^8^ Thus, to ensure proper activity and the quality, safety, and efficacy needed to gain regulatory approval, a candidate mAb requires extensive analytical testing at high levels of sensitivity.

To prove similarity to the reference molecule, biosimilar candidates are subjected to rigorous characterizations.^9^ If the biosimilar's characteristics satisfactorily match those of the reference product, a reduced number of clinical studies may be needed, potentially reducing costs and expediting its entry onto the market. To demonstrate molecular similarity, regulatory guidelines have been established.^10-11^ Suggested common analysis include amino acid sequence and composition, peptide map, disulfide bonds and sulfhydryl characterization, molecular mass, isoform patterns, and glycan profiles.^12^ The overall structural characterization must also demonstrate that the biosimilar mAb's higher-order structure (HOS) closely mimics that of the reference product. Additionally, HCPs also need to be analyzed to demonstrate the comparable impurity concentration. One powerful analytical method to assess the overall similarity between biosimilar and reference products at the molecular level is ultra-performance liquid chromatography-mass spectrometry (UPLC-MS), which has proven to be reliable and have sufficient throughput.

Previously, our group described a series of UPLC-MS methodologies to assess the “first layer” of similarity between Herceptin® (trastuzumab) and a candidate biosimilar.^13^ Inflectra® and Remsima® are the first biosimilar versions of Remicade® (infliximab) to be approved for use in Europe,^14^ South Korea^15^ and the US. This biosimilar mAb product is thus the first model available to assess the utility of UPLC/MS technologies for probing the HOS, glycosylation profiles, and HCP impurities of a molecule that is considered a biosimilar by both the European Medicines Agency and the Food and Drug Administration. As a complement to a previous biosimilarity report,^16^ in-depth analyses are presented here pertaining to the HOS using hydrogen-deuterium exchange mass spectrometry (HDX MS), host cell proteins (HCP) analysis via 2D UPLC-IMS-MS, and a rigorous analysis of the glycan profiles facilitated by Glycoworks™ *Rapi*Fluor-MS™ to demonstrate the similarity between infliximab reference (Remicade®) and a biosimilar (Inflectra®) product.

## Results

### Glycan profile analysis

To assess the similarities and possible differences between the reference and biosimilar products, a released N-glycan analysis was performed. The method used in this study is founded upon derivatizing glycans using a novel reagent, Glycoworks™ *Rapi*Fluor-MS™. This molecule has been shown to increase both fluorescence (FLR) and MS sensitivity levels,^17^ and to facilitate the detection of lower abundance-level glycans.

In this investigation, a total of 23 mass-confirmed glycans with relative abundances greater than 0.2% were identified on the reference mAb, while 21 of these were determined to be on the biosimilar product. Two glycans were unique to the reference molecule. The glycans found on each molecule are summarized in Table 1, along with their abundance levels and the percent differences for the biosimilar molecule. (The percent difference was calculated using the average normalized abundance values for the reference molecule).

**Table 1.** A comparison of the mass-confirmed glycans present at normalized abundances greater than 0.2% for innovator and biosimilar infliximab. In this report, we use the following glycan nomenclature: F- Fucose; G- Galactose; Sg- N-glycolylneuraminic acid, Ga- α-linked Galactose; A1- Monoantennary, A2- Biantennary. Numbers with parentheses indicate the preceding monosaccharide's linkage while those not in parentheses indicate the preceding characteristic's number. For example, F(6)A3G2Ga1Sg1 represents a core fucosylated triantennary glycan with 2 galactoses directly attached to antennae, 1 galactose linked via an α linkage, and one antenna terminated with an N-glycolylneuraminic acid. Symbols: blue square- N-acetylglucosamine; green circle- mannose, yellow circle- galactose, red triangle- fucose; gray diamond- N-glycolylneuraminic acid.

**Figure 1.**
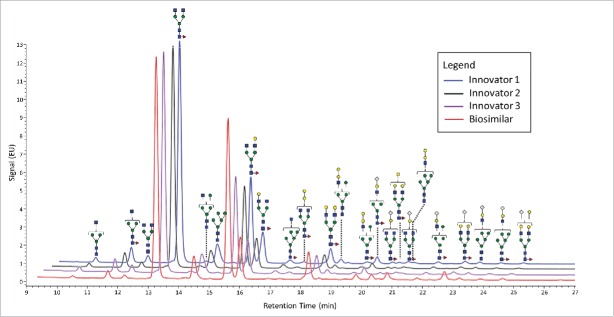
FLR chromatograms of released N-linked glycans from 3 innovators batches of infliximab (blue, black, and purple traces) and one batch of biosimilar (red trace). The symbols are the same as those used in Table 1.

The overall qualitative profiles for both the reference and biosimilar mAbs are very similar, and are in general agreement with a previous study.^16^ Fig. 1 presents stacked FLR chromatograms annotated with glycan structures and shows the general similarities between the different molecules. This figure demonstrates that there are 6 higher abundance glycans (defined as fluorescence absorbance values of ∼2 or greater) and a number of lower abundance structures. All three major classes of N-linked glycans were observed: complex-type, high mannose, and hybrid structures. A total of 8 acidic glycans were observed, and the remainders were neutral. In this study, no bisecting structures could be confirmed.

Quantitatively, Fig. 1 shows that glycans with higher abundance levels (∼2 absorbance units and higher), were quite comparable for both mAbs, with the exception of F(6)A2[6]G(4)1 (refer to Table 1 for glycan structures and the nomenclature used in this report). The majority of the more pronounced changes were observed for the lower-abundance glycans, those recorded at 0.5 absorbance units or less for the FLR signals, which roughly corresponded to normalized abundances of 1% or less. Interestingly, these differences appeared to be associated with different classes of glycans and were generally unique based on the origin of the mAb. For example, the α-galactosylated glycans, those lacking galactoses, (F(6)A2, F(6)A1, A2, A1, F(6)M5A1, and F(6)M4A1) were all determined to be present at decreased abundance levels on the biosimilar mAb. These differences ranged from −18% to −66%. Conversely, the galactosylated species that did not contain α-linked galactose pairs (F(6)A2G(4)2 and F(6)A2[6]G(4)1) were observed to be elevated in their abundance levels by about 50% on the biosimilar mAb. Interestingly, the normalized amount of F(6)A2[3]G(4)1 did not appear to have significantly different relative abundance levels on either molecule.

**Figure 2.**
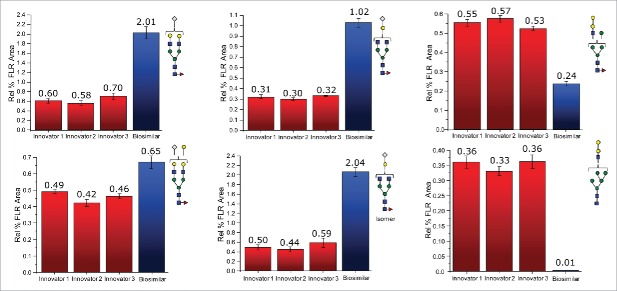
Differences in the normalized relative intensities (based on FLR data) for selected glycans featuring immunogenic glycans.

For mAbs grown in non-human cell lines, glycans terminated with N-glycolylneuraminic acid and those containing galactose pairs connected via an α-type linkage are of substantial interest. These glycans are known to induce a severe immunogenic response in many individuals. As seen with the other classes of glycans, each of these 2 types seemed to be preferentially expressed on a particular mAb. A total of 8 glycans were terminated with N-glycolylneuraminic acid, and 6 of these were observed to be elevated on the biosimilar molecule. F(6)A2G(4)1Sg1, F(6)A2G(4)1Sg1 iso, and F(6)A2G(4)2Sg(6)1 were elevated between 200 and 300% relative to the reference mAb, while F(6)A2G(4)2Ga(3)1Sg(6)1, F(6)M5A1G(4)1Sg(6)1, and M5A1G(4)1Sg(6)1 were elevated but to a lesser extent. F(6)M4A1G(4)1Sg(6)1 and F(6)A1G(4)1Sg(6)1 were observed to be slightly decreased in their abundance levels on the biosimilar mAb. In this study, we also identified 6 glycans featuring an α-linked galactose-galactose pair, and 5 of these glycans, (A2G(4)1Ga(3)1, F(6)A2G(4)2Ga(3)1, M4A1G(4)1Ga(3)1, M5A1G(4)1Ga(3)1, and F(6)M4A1G(4)1Ga(3)1), were more abundant on the reference molecule. Two of these glycans, M5A1G(4)1Ga(3)1 and F(6)M4A1G(4)1Ga(3)1, were only identified on the reference mAb. Interestingly, the only glycan with α-linked galactoses that was elevated in its abundance level on the biosimilar mAb also was terminated by an N-glycolylneuraminic acid. Fig. 2 presents bar graphs for several glycans with more pronounced differences between the 2 different mAb samples.

Fucosylation is another monosaccharide of interest associated with mAb biopharmaceutical products. Fucose influences a number of properties, including serum half life and the cytotoxicity of the drug.^18^ Both mAbs had very similar levels of fucosylation overall; the reference mAb's level of fucosylation was determined to be 90%, while the biosimilar's fucosylation level was 95%.

### Hydrogen deuterium exchange (HDX) analysis

HDX MS is a powerful technique for the HOS analysis of proteins. By monitoring the exchange behavior of a protein's backbone amide hydrogen, the technique works as an indicator of solvent exposure and hydrogen bonding, which provides information about the HOS of a protein. Here, the deuterium incorporation as a function of time for each peptic peptide was determined for all 4 infliximab samples (3 batches of the reference product and one biosimilar batch). The result was acquired from triplicate experiments. A total of 191 heavy chain peptides and 89 light chain peptides were commonly identified in all of the reference and biosimilar samples, which covers 100% of the amino acid sequence for all of the 4 samples (Fig. S1).

**Figure 3.**
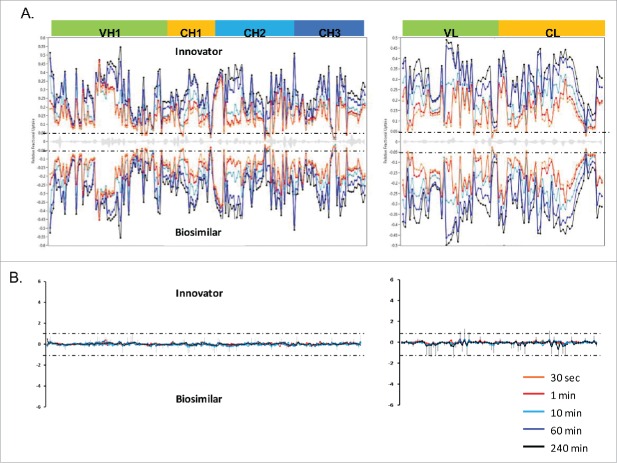
HDX MS deuterium exchange mass spectrometry comparability profile of the biosimilar and one of the innovator samples. (A) A butterfly plot of the average relative fractional exchange data for Innovator (top) versus biosimilar (bottom), as a function of peptide order. The x-axis is the calculated peptide midpoint. The y-axis is the average calculated relative fractional exchange. The orange, red, cyan, blue, and black lines correspond to data acquired at 30 s, and 1, 10, 60, and 240 min of deuteration, respectively, for both samples. The standard deviation between 2 measurements across all peptides was less than ±0 .05 Da, which is shown in the middle in gray. (B) Differential Plot.

**Figure 4.**
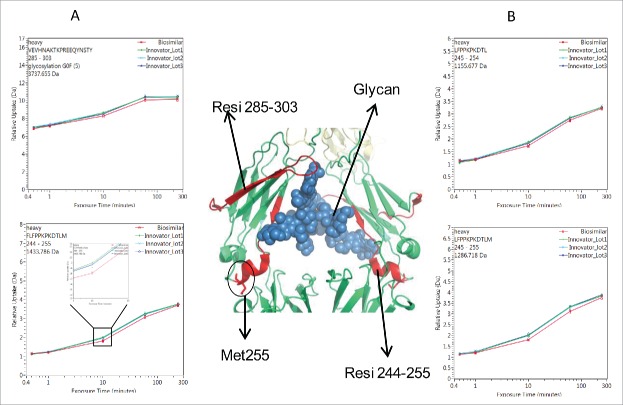
Comparison of deuterium incorporation of innovator and biosimilar samples. (A) Representative deuterium incorporation profiles of regions (residues 244–255, 245–254, 245–255, 285–303) in CH2 domains shows a minute difference. The red line represents the data from the biosimilar product, the green, cyan and blue lines represent the data from the 3 batches of the innovator samples. The experiments have been repeated in triplicate runs. (B) The location of the region that displayed minor difference among biosimilar and innovator samples are colored in red in the model structure of IgG1 (PDB: 1HZH). Glycosylation is shown in blue. Met255 is circled and shown in stick notation.

**Figure 5.**
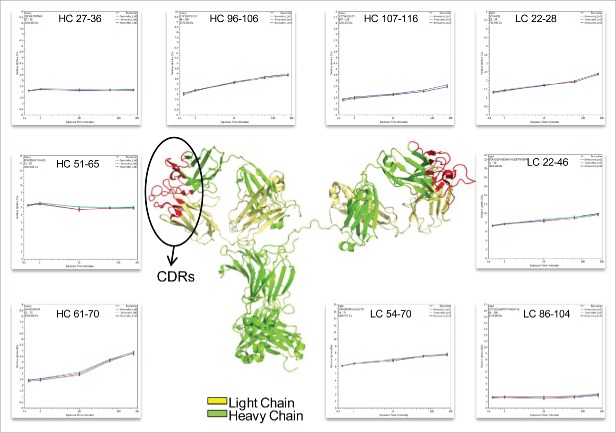
Representative peptides covered the complementarity determining regions (CDRs) of infliximab displayed identical conformation and dynamics. The heavy chain and light chain structures are colored in the 3D model of IgG1 (PDB: 1HZH) in green and yellow, respectively. The three light and heavy chain CDRs are colored in red. The deuterium incorporation curves of the sample peptides, which covered all the CDRs, are showed.

The deuterium incorporation of these peptides for each sample was analyzed and compared. Overall, no distinct differences in deuterium incorporation between reference and biosimilar product samples were observed as shown in the butterfly plot in Fig. 3A and in the heat map graphs of Fig. S2. This agrees well with a prior study comparing the HOS between these 2 mAbs using circular dichroism and Fourier transform infrared spectroscopy.^16^ Besides global structural information, HDX MS provides detailed information on the locations of differences in conformation. According to a previous publication,^19^ it is considered to be a significant difference in deuterium levels if the mass difference (Δm) is different by ± 0.5 Da at any time point or if the summation of any difference found in the peptides differs by ± 1.1 Da during the course of deuterium incorporation. No peptides displayed changed deuterium incorporation outside the statistically determined significance threshold (dotted lines in differential plot in Fig. 3B) from this study. This indicates that, in terms of the tertiary structure, the biosimilar sample is very comparable to the reference product. However, there were a few peptides in the CH2 domain that attracted our attention due to their minute, but consistent, differences between the biosimilar and reference products. These differences were confirmed by multiple overlapping peptides and are shown in Fig. 4. Our ability to detect these slight differences was due to the high reproducibility of our method and low standard deviation (± 0.05 Da, shown in Fig. 5 in the gray box). These differences were confirmed by multiple overlapping peptides. Fig. 4 shows the exchange profiles of the 2 segments (residues 244–255 and residues 285–303) in the Fc-CH2 domain.

One of the noted differences covers residues 244–255, which is located at the N-terminus of the CH2 domain. Compared with the reference molecule, the biosimilar mAb displayed slightly decreased deuterium incorporation (Fig. 4). The differences in deuterium uptake amounts among these samples were small (∼0.2 deuteron per peptide) and not considered significant.^19^ However, the difference was measurable and confirmed by overlapping peptides. In Fig. 4, 3 representative peptides (244–255, 245–254, and 245–255) displayed the same deuteration profiles. Taking 244–255 as an example, following 10 and 60 min of sample exposure to D_2_O, the biosimilar was less deuterated by 0.2 Da than the reference mAb. This indicates that the sub-region 244–255 in the biosimilar product adopts a conformation that was less prone to exchange with the solvent. For longer incubation periods, the differences disappeared, most likely because this area eventually becomes exposed to solvent due to protein native movement. Based on the crystal structure, the sub-region 244–255 contains a less stable small α-helix and a loop, which indicates a relatively loose conformation that is sensitive to external disturbances.

Interestingly, the region with slightly more conformational protection in the biosimilar sample that we identified in this study is similar to the regions that become more flexible when an immunoglobulin (Ig)G is oxidized,^20-21^ those that are glycosylated,^21^ or those where a high level of high mannose-type glycans are found.^22^ Thus, these slight differences could be caused by methionine oxidation or glycosylation profile changes. To confirm which factor is responsible for the observed changes, a peptide mapping experiment was performed to determine the oxidation level among the different samples. A normalized label-free quantitation technique was used to compare the abundance of the oxidized peptide based on ion intensities

In order to obtain meaningful data on oxidation, it is crucial to minimize oxidative artifacts caused by sample preparation. To detect and quantify the site-specific Met sulfoxide, the mAb samples were digested with trypsin and then analyzed via reversed-phase (RP) UPLC-MS. The biosimilar sample yielded a chromatogram highly similar to that of the reference product sample (Fig. S3), showing that there was no significant modification difference.

The oxidation level of each Met residue was determined based on the MS signal intensity of the Met-containing peptide T22 (amino acid sequence: DTLMISR), and is shown in Table S1. Met 255 did not exhibit diastereomers of methionine sulfoxide, as previously reported.^23^ Only one peak containing an oxidized Met255 was taken into consideration. A label-free quantitative LC/MS^E^ approach^24^ was used to characterize the oxidation level in the samples. The oxidation levels for the T22 peptide for all of the 4 samples were highly comparable and were all less than 3%, suggesting Met oxidation was unlikely to be responsible for the conformational change observed in the HDX-MS experiment.

Another region that shows a deuterium uptake difference among the samples spans the residue containing the site of N-linked glycosylation. These peptides exhibited the same deuterium uptake pattern as the region 244–255, (see Fig. 4, peptide VEVHNAKTKPREEQYNSTY (285–303) with the F(6)A2 glycan). After 10 and 60 min exposure times to D_2_O, a slight decrease (∼0.2 Da) in deuterium uptake was found. This difference was no longer detectable after the labeling time was increased to 4 h.

The location of the peptides that displayed conformational differences is shown in the crystal structure of IgG1 (Fig. 4) and is highlighted in red. Glycans attached to position N-300 are shown as blue spheres. The illustration indicates that the differences in the glycosylation profiles at N-300 may be a contributing factor causing the conformational change.

### The HDX level of CDR regions

The complementarity-determining region (CDR) is critical to the specificity of mAbs. The highest degree of variability between different mAbs exists in 3 small CDR sub-regions within the variable domains of the light and heavy chains.^25^ The native structure of the CDRs of the light and heavy chains form a cleft that serves as the antigen-binding site of an IgG.^26^ Because the amino acid sequences of the CDRs determine the shape and ionic properties of the antigen-binding site, the CDRs define the specificity of the antibody. As reported previously, proteins with similar structures may display different dynamics, which affects their biological functions.^27^ In our study, the peptides covering all the CDRs of infliximab (HC: 26–37; 52–70; 103–116, and LC: 24–39; 55–61; 94–102)^28^ were selected and compared for deuterium incorporation levels (Fig. 5). This comparison indicates that the biosimilar and reference mAbs share the same deuterium behavior, which suggests that there is no difference in their ability to bind the antigen.

### Host cell protein analysis

We applied a recently developed HCP assay^29^ to make a direct comparison of the HCP profiles for the reference infliximab product and its biosimilar version. The HCP assay relies on 5 steps: 1) proteolytic digestion of mAbs; 2) 2-dimensional separation of the mAb proteolytic peptides by high pH/low pH RP/RP UPLC; 3) electrospray ionization (ESI) coupled with ion mobility separation (IMS) of peptide precursors followed by high resolution mass spectrometric detection; 4) drift-time specific fragmentation of peptide precursors using a quasi “fixed” collision energy;^30-31^ and 5) database searching for HCP identification and quantification based on several spiked-in protein digest standards.

The reference mAb and its biosimilar were digested and analyzed in triplicate using the 10-step 2D-LC high-pH RP/low-pH RP HDMS^E^ assay described in the Experimental Section. Four unique protein digest standards, yeast alcohol dehydrogenase (ADH), rabbit phosphorylase b (PHO), bovine serum albumin (BSA), and yeast enolase (ENL), originating from species other than the murine cells used for mAb expression, were spiked into the reference and biosimilar product digests post-digestion. The standard protein digests, spiked at different concentration levels, were used as internal calibrants to probe the dynamic range of the assay, and for the measurement of each individual HCP using the summed signal of the 3 best-responding peptides for each protein.^24,32^ The individual HCP amounts (expressed in femtomoles) were calculated against the top 3 ESI-MS responding peptides from PHO (one of the protein calibrants) loaded at 1,000 femtomoles on-column.

**Figure 6.**
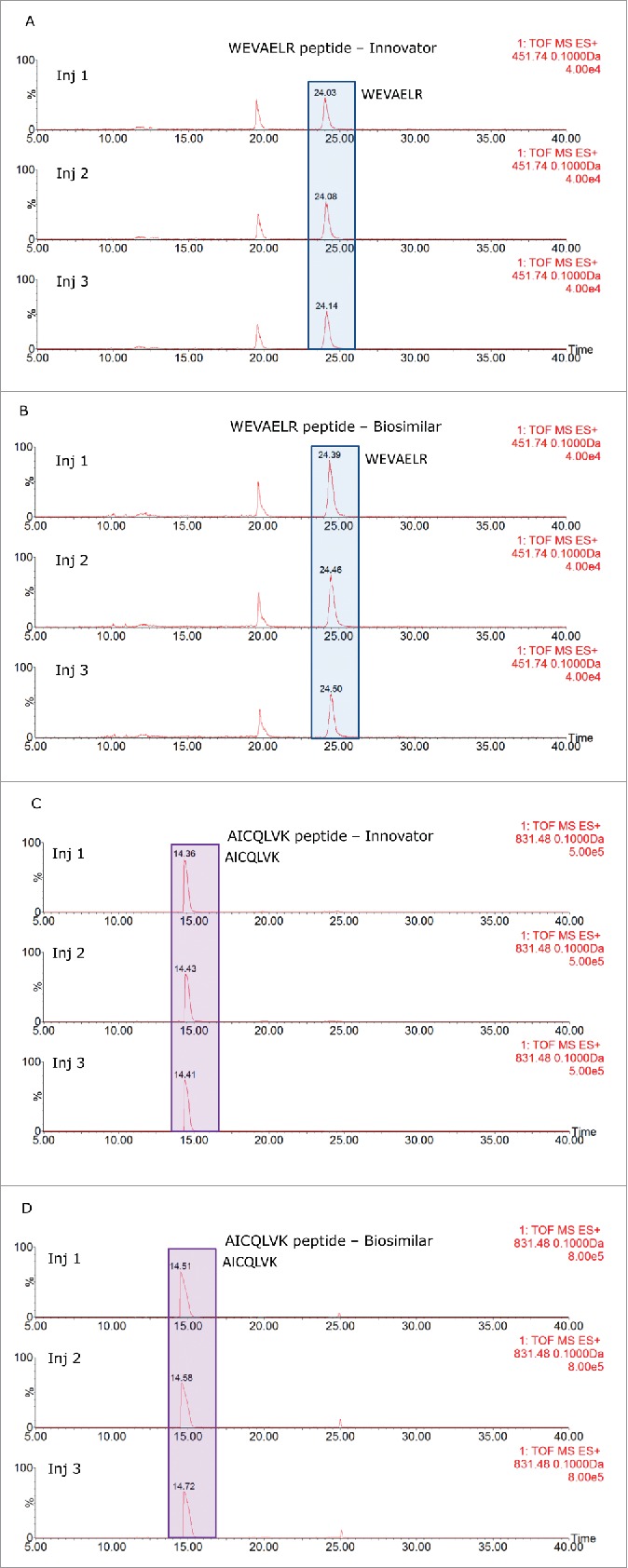
Extracted mass chromatograms for WEVAELR from HCP Epidermal growth factor-like protein 8 for the innovator (A) and biosimilar (B) infliximabs and AICQLVK from WD repeat-containing protein 37 from the innovator (C) and biosimilar (D) products. Each sample was injected in triplicate.

**Figure 7.**
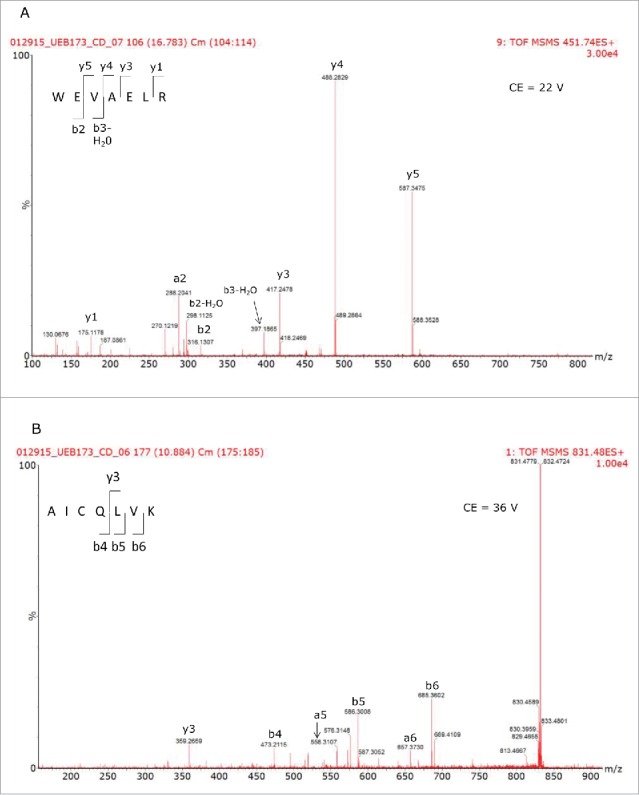
Tandem MS spectra used to confirm the sequences of the peptides used to identify the HCPs present in the different mAb samples.

The results of the 2D-LC/HDMS^E^ analysis are displayed in Table 2A and 2B for the reference and biosimilar mAbs, respectively. Two murine proteins (epidermal growth factor-like protein 8 and WD repeat containing protein 37) were identified as HCPs in both mAbs, along with all of the spiked-in proteins. The individual HCP concentrations, calculated from 3 replicate injections, indicated that they were present at levels about 2 times higher in the biosimilar product. Fig. 6A–D shows the mass chromatograms obtained for 2 HCP peptides (WEVAELR epidermal growth factor-like protein 8 from and AICQLVK WD repeat containing protein 37) across all of the replicates recorded for both mAbs. In comparison, Fig. 6A–B reveals that WEVAELR is about twice as abundant in the biosimilar sample. These mass chromatograms are given on the same raw intensity scale and the reference mAb was observed at an intensity of about 2e4, while the biosimilar was present at an intensity of about 4e4. Fig. 6C–D presents mass chromatograms from the peptide AICQLVK. These show that this peptide was present at elevated abundances in the biosimilar sample. Confirmatory MS/MS spectra of these peptides are shown in Fig. 7A and 7B for WEVAELR and AICQLVK, respectively. These tandem MS spectra were obtained after re-analyzing the 2 mAb digests with the 10-step 2D-LC separation. To confirm the results of the prior discovery experiments, we conducted targeted MS/MS experiments. In this design, 3 unique steps were performed. The first of these was quadrupole filtering of peptide precursors, followed by a subsequent ion mobility separation, and then fragmentation in the transfer cell of the Synapt G2-S instrument using a fixed, optimized collision energy. This method eliminated fragment ions of co-eluting peptides from appearing in the spectra of the analytes of interest. These “clean” MS/MS spectra provide additional confirmatory sequence information for both peptides. Even though both peptides are relatively short (7 amino acids), they have very unique sequences among the proteins from the mouse proteome. Their uniqueness was confirmed by a BLAST search (http://blast.ncbi.nlm.nih.gov/Blast.cgi) against the 16,648 proteins from the mouse proteome (UniProt database).

**Table 2.** A comparison of host cell proteins between innovator (A) and biosimilar (B) infliximab.

## Discussion

In this study, we focused on 2 main areas of mAb analysis used for comparative purposes: structure characterization and impurities present in the final products. We analyzed the samples using seemingly unrelated experimental designs in order to tackle analytical challenges that are important in biosimilar development/assessment, and for which no well recognized methodologies have been widely accepted. These challenges include the assessment and objective comparison of HOS, minor glycans, and HCP profiles between a reference product and its biosimilar. The unifying theme is our reliance on sensitive analytical techniques/methods that are capable of delivering key information needed before decisions regarding biosimilarity can be confidently made. Using *Rapi*Fluor-MS™, we were able to successfully determine the structures of several low abundance glycans that were problematic using other labeling techniques, including the commonly used 2-aminobenzamide. This is potentially very important as the immunogenic glycans were observed at low abundance levels, and the trace-level glycans were generally the ones that we observed to be at different abundance levels and could be at least partly responsible of the slight alterations that we observed in the HOS of the 2 mAb samples. In order to observe these minor alterations to the structure, highly reproducible methods were needed to ensure the validity of the experimental results. Hydrogen deuterium exchange coupled with high resolution and high mass accuracy mass spectrometry monitors deuterium incorporation, enables precise and sensitive data collection, and provides insight on local environment change of amide hydrogen, and thus it can be used to effectively assess the HOS similarity between the reference product and its biosimilar. Likewise, a highly sensitive method that is capable to identify the nature of HCPs is needed to characterize host cell protein impurity profile. This method should be different from traditional ELISA assays for HCP measurement, which only provides a measure of total concentration (with no identity information) of a range of HCPs that are immunoreactive to the ELISA reagent. Use of this type of ELISA makes it difficult to compare the HCP measurement results from a reference product and its biosimilar counterpart because the exact assay methods and the related reagents used by the companies that developed the mAb products tend to be proprietary, and thus are likely not the same. A LC/MS approach that can identify and quantify HCP in a final drug substance offers a fair and more precise means to assess the HCP profile in both reference and biosimilar samples. In this work, we observed low abundance HCPs at concentrations of a few hundred ng/mL when the mAb is at 10 mg/mL. Additionally, like the HOS analysis, the HCP assay provides more detailed information on the nature of HCPs and its distribution. Nonetheless, identification of HCPs from drug substance requires exquisite instrumental methods to ensure the HCP peptides were fragmented, and thus correctly identified and quantified. The 2D-LC-IMS MS method employed in this work takes advantage of the substantial separation power offered by the 2D chromatography and IMS to resolve the low abundance of HCP peptides from the overwhelming amount of peptides from drug proteins, rendering a greater chance for fragmentation of the low intensity ions from HCPs.

Only four product batches (3 references and one biosimilar) were analyzed in this study. We do not claim that this sample set is sufficient to perform a “true” biosimilar comparison exercise. Instead, the results presented here are our initial attempt to demonstrate the utility of the methodologies and the capability of the techniques to discern structural/impurity changes at high sensitivity levels so these quality attributes can be fairly monitored and compared in real biosimilar development processes. The validity of our methods is manifested by the close correlation of our results with a previous report analyzing many more samples using alternative techniques.^16^ More importantly, the data presented here render insights on the samples that the previous work did not provide, i.e., the detailed examination for HOS and HCP profile. Both of the attributes are important to control in biosimilar product development, yet the current analytics for the attributes typically struggle to provide insightful information to aid development. The methods we present here have sufficient throughput to be deployed for larger-scale studies. The valuable information gained from the technologies in the current study suggests that deployment of such methodologies can greatly aid the advanced characterization of mAb samples and facilitate the development of mAb biosimilar products.

## Methods and materials

### Materials

Remicade®/infliximab (Janssen Biologics, The Netherland) and Inflectra®/infliximab (Celltrion, South Korea) were purchased from a pharmacy located in the European Union. Ammonium bicarbonate, ammonium hydroxide (28% w/w), LC-MS grade water, sodium chloride, guanidine hydrochloride (GdnHCl), sodium phosphate monobasic, sodium phosphate dibasic, and MS-grade formic acid (FA) were purchased from Sigma Chemical Co. (St. Louis, MO, USA). HPLC-grade acetonitrile (ACN) and tris(2-carboxyethyl) phosphine (TCEP) were obtained from Thermo Fisher Scientific (Rockford, IL). A Milli-Q Elix-3 purification system (Millipore, Bedford, MA) was used to prepare the deionized (DI) water (18 MΩ• cm) required for all experiments. Protein digestion standards of yeast alcohol dehydrogenase (ADH), rabbit phosphorylase b (PHO), bovine serum albumin (BSA), and yeast enolase (ENL) (MassPREP digestion standards) were products of Waters Corp. (Milford, MA) along with the pepsin column and the complete Glycoworks™ *Rapi*Fluor™ kit. (This kit contained all components needed for sample preparation.) Deuterium oxide (99.9%) was acquired from Cambridge Isotope Laboratories (Tewksbury, MA). Trypsin and Lys C were obtained from Promega, (Madison, WI).

### Methods

#### Glycan profiling

**N-Glycan Release**. N-Glycan samples were prepared according to the procedures described in the Care and Use Manual for *Rapi*Fluor-MS™ (RFMS). Briefly, mAb samples were diluted to a concentration of 2 μg/μL and a 7.5-μL aliquot was added to 15.3 μL of water and 6 μL of a 5% Rapigest™ solution. The mAbs were denatured thermally at 95°C for 5 min. After cooling to ambient temperature, a 1.2-μL aliquot of Rapid PNGase F was added to release the N-linked glycans, as their glycosylamines, through a 5-minute incubation at 55°C. Each sample was prepared in triplicate.

**Glycan Labeling**. Following their release as glycosylamines, the glycans were labeled with RFMS (96 sample reaction kit). The reagent (23 mg) was dissolved in 335 μL of anhydrous dimethylformamide (DMF) and a 12-μL aliquot of this solution was added to each glycan sample and allowed to react at ambient temperature for 5 minutes. Subsequently, a 358-μL aliquot of ACN was added to prepare the samples for hydrophilic interaction liquid chromatography (HILIC)-based purification.

**Glycan Purification**. Derivatized N-linked glycans were purified using a HILIC μ-Elution plate. The medium was first washed 3 times with 300-μL aliquots of water, followed by three 300-μL aliquots of an 85%/15% ACN/water solution. The glycan-containing samples were then loaded onto the medium and subsequently washed 3 times with 600-μL aliquots of a 90%/9%/1% solution of ACN/water/FA. The glycans were eluted by three 30-μL aliquots of SPE Elution Buffer (200 mM ammonium acetate in 5% ACN).

**UPLC-MS**. Following purification, the glycans were diluted with 100 μL of DMF and 210 μL of ACN to prepare them for HILIC UPLC-MS analyses. A Waters Acquity H-class Bio UPLC system, comprising a solvent manager, a sample manager, operating at 5°C, a column manager, maintained at 60°C, and a FLR detector coupled to a Waters XEVO G2-XS QTof MS, was used to analyze the samples. The instrument system was controlled by UNIFI. Glycans were separated with a Waters UPLC Glycan BEH Amide column (2.1 × 150 mm, 1.7 μm particle size, 130 Å pore size) with Mobile Phase A being a 50 mM ammonium formate solution (pH = 4.4) and Mobile Phase B was ACN. Separation was achieved by gradient elution conditions ranging from 75%-54% Mobile Phase B over 35 minutes at a flow rate of 0.4 mL/min. Glycans were detected at 265 nm (excitation)/425 nm (emission) using the FLR detector and subsequently further mass analyzed over the *m/z* range spanning 500–2000.

**Data Analysis**. UPLC/FLR/MS data were processed and analyzed using the Glycan Assay (FLR with MS confirmation) workflow in UNIFI. This workflow first converted the retention time of the mAb glycan samples to glucose units (GU) based on a calibration curve of dextran labeled with RFMS. These data were then used for GU library searching for glycan identification, which were then mass confirmed using MS data. (If ambiguous library searches resulted, the correct identification was confirmed with tandem MS information.) The library searches used a GU tolerance of 0.2 GU and a mass error of 0.01 Da. Glycan abundances were reported as normalized values, where the FLR peak area for each glycan were expressed as a percentage of the total summed peak area for all glycans identified.

#### HDX analysis

**Sample Preparation**. Three biological lots of the reference product and one biosimilar sample were prepared by diluting the protein stock solutions (∼12 pmol/µL) 15-fold (v/v) with equilibrium buffer (50 mM sodium phosphate, 100 mM NaCl in H2O, pH = 6.8). Labeling was initiated by diluting the protein stock solution with a labeling buffer (50 mM sodium phosphate, 100 mM NaCl in D2O, pD = 6.4. After labeling, the reaction was quenched with an equal volume of pre-chilled 200 mM sodium phosphate buffer with 0.5 M TCEP, 4 M GdnHCl, pH = 2.3. The quenched samples were injected onto a Waters M-class UPLC with HDX Manager (Waters Corp.). The sample preparation, including deuterium labeling, quenching, and peptic digestion, was performed on LEAP PAL3 system (LEAP Technologies, Carrboro, NC and controlled by Chronos software (Axel Semrau, Germany).

**UPLC and MS Analysis**. The protein samples were digested online using a BEH immobilized pepsin cartridge (dimensions 2.1 × 30 mm) (Waters Corp.).^33^ All the chromatographic elements were held at 0.0 ± 0.1°C in the cooling chamber for the entire time of the separation. The injected peptides were trapped and desalted and then the separation conditions were optimized. Deuterium levels were not corrected for back exchange and are therefore reported as relative. However, all comparison experiments were carried out under identical experimental conditions thus negating the need for back exchange corrections.^34^ All experiments were performed in triplicate. The error of determining the deuterium levels was ± 0.05 Da in this experimental setup. To eliminate peptide carryover,^35^ a wash solution of 1.5 M GdnHCl, 0.8% FA, and 4% ACN was injected after each run.

Mass spectra were obtained with a Synapt G2-S equipped with a standard ESI source. Mass spectra were acquired over an m/z range of 100–2000. The peptic identification list was generated by PLGS 3.0 (Waters Corp, Milford, MA, USA) using a combination of exact mass and MS^E^ fragment data. Deuterium exchange data were processed with DynamX 3.0 (Waters Corp.). PyMOL was used to map the conformational changes on the crystal structure of an IgG1 antibody (PDB: 1HZH).

**Reduced peptide mapping with mass spectrometry and label-free quantification**. The antibody samples were digested with trypsin by first diluting them with a denaturing buffer containing 8 M GdnHCl and 0.225 M tris(hydroxymethyl)aminomethane, pH = 7.5, to a final concentration 1 mg/mL. The samples were then incubated with 0.5 M dithiothreitol (DTT) for 30 min at 37°C. Alkylation was done by adding 0.5 M iodoacetamide and incubating the samples at 25°C for 15 min; the reaction was then quenched by adding 0.5 M DTT. The samples were buffer exchanged using NAP-5 columns (GE healthcare) into 0.1 M Tris buffer (pH 7.5). The digestion was performed with the addition of 20 µg of trypsin at 37°C for 1 hr.

All protein digests were analyzed with an ACQUITY UPLC BEH C_18_ column (1.7 μm, 2.1 mm × 100 mm column, Waters Corp.) A Waters UPLC H-class coupled to a Waters Xevo G2-XS QTof MS was used. For these separations, Mobile phase A was aqueous 0.05% (v/v) trifluoroacetic acid (TFA) and Mobile phase B was 0.05% (v/v) TFA in ACN. Peptides were eluted from the column with a linear gradient ranging from 0.5% to 40% B over 90 min at 60°C. Trypsin digestion of reference and biosimilar product samples resulted in over 97% amino acid sequence coverage. Oxidized Met peptides were identified and quantified using UPLC-MS^E^ data using UNIFI.

#### HCP analysis

**Preparation of Ammonium Formate (NH**_**4**_**FA, pH 10) Solution**. A stock solution of ammonium formate (200 mM, pH = 10) was prepared by mixing 6.95 mL of 28% (w/w) ammonium hydroxide with 450 mL of deionized water. Then, 0.81 mL of FA was added to the solution. The pH of the stock solution was adjusted to 10 with FA, and the final volume was brought to 500 mL. The stock solution was diluted (1:10, v/v) using deionized water to yield a 20 mM NH_4_FA solution for sample preparation and 2D-LC separations.

**Digestion of mAb samples.** One milligram of each mAb (contained in 50 µL of 20 mg/mL Remicade® and in 100 µL of 10 mg/mL Inflectra®) was denatured with 0.05% *Rapi*Gest SF (in 50 mM ammonium bicarbonate) for 15 min at 60°C, reduced with 20 mM DTT for 60 min at 60°C, alkylated with 10 mM iodoacetamide (IAM) for 30 min (at room temperature) and digested overnight (37°C) with a mixture of porcine trypsin and Lys C enzymes using a 1:20 molar ratio (enzyme: protein). After digestion, the RapiGest SF surfactant was decomposed by adding 5 µL of FA, and the samples were incubated for 30 min at 37°C and centrifuged (15 min at 12,000 rpm) to separate the insoluble component of RapiGest SF by precipitation. The supernatant was transferred to a new vial, and the pH of the solution was adjusted to ∼ pH = 9 by adding 25 µL of 25% ammonium hydroxide. Four Waters MassPREP protein digests standards (20 µL of 1 µM ADH, 40 µL of 100 nM PHO, 10 µL of 100 nM BSA and 20 µL of 10 nM ENL) were spiked in the mAb digests, and the total digest volume was brought to 1 mL using 20 mM ammonium formate (pH = 10). While the total amount of the mAb digest loaded onto the first dimension column was ∼250 µg, with an injection volume of 250 µL, the loaded amounts of the spiked protein digests were 5,000 fmoles of ADH, 1,000 fmoles of PHO, 250 fmoles of BSA and 50 fmoles of ENL.

**2D-LC**. The 2D-LC separations were performed on an ACQUITY UPLC^®^ M-Class UPLC^®^ system (Waters Corp.) equipped with online dilution technology, as previously described.^29^ The first chromatographic dimension of peptide fractionation was performed under basic (pH = 10) conditions on a BEH C_18_ 300 Å, 5 µm 1.0 mm × 50 mm RP column (XBridge, Waters Corp.). These fractionations were performed at 60°C and a flow rate of 10 µL/min. Eluent A was aqueous 20 mM ammonium formate (pH = 10) and eluent B was neat ACN. The step elution gradients for the first dimension were optimized such that approximately the same amount of peptides was eluted off at each step. Both mAb digests were analyzed using a 10-step fractionation method. These fractions were eluted from the first dimension using compositions of 10.8, 12.4, 14.0, 15.4, 16.7, 18.6, 20.4, 25.0, 30.0 and 50% Eluent B, respectively. The fractionation process was programmed to start immediately after the completion of sample loading (20 min at 10 µL/min with 3% B). Each first dimension elution step was performed with a 20 min run time using a flow rate of 10 µL/min. Eluted peptide were mixed on-line with 90 µL/min of 0.1% TFA solution (1:10 dilution) before being trapped on the trapping column (300 µm × 25 mm, packed with 5 µm 100 Å silica-based C_18_ (Symmetry, Waters Corp, Milford, MA). The mobile phases for the second chromatographic dimension (low pH RP) were 0.1% FA in water (mobile phase A) and 0.1% FA in ACN (mobile phase B). The second dimension column was a 0.3 mm × 150 mm C_18_column packed with CSH (charged surface hybrid) 1.7 µm particles (ACQUITY UPLC M-Class CSH C_18_, Waters Corp., Milford, MA). The flow rate for the second dimension separation was 10 µL/min, while the column was maintained at 60°C. A 40-min gradient from 3 to 40% B was used for separating peptides in the second separation dimension. The column was then washed using 90% B for 1 min and re-equilibrated at 3% B for 7 min before returning to the next step of fractionation.

**Mass spectrometry**. A data-independent acquisition method (HDMS^E^) was employed for the identification and quantification of HCPs (discovery assay). LC/HDMS^E^ data were acquired using a traveling wave ion mobility enabled quadrupole time-of-flight mass spectrometer (SYNAPT G2-S, Waters Corp., Milford, MA) equipped with the standard electrospray ionization (ESI) probe fitted with a small bore (45 µm ID) stainless steel capillary. For HCP validation, “pure” MS/MS spectra were acquired after several targeted peptide precursors were isolated using the quadrupole mass filter, separated from other co-eluting precursors using ion mobility, and fragmented with an optimized (fixed) collision energy in the transfer-cell. For all measurements, the mass spectrometer was operated in positive ion resolution mode, with a typical resolving power of 20,000 FWHM. Data were acquired in continuum mode over *m/z* range of 100–1990, using a capillary voltage of 2.6 kV, a source temperature of 100°C, a source offset voltage of 100V, a cone gas flow of 50 L/h and a cone voltage of 40 V. The desolvation temperature was set to 250°C and the desolvation gas flow rate was 500 L/hour. The LC/HDMS^E^ data were collected by alternating the collision energy of the transfer cell between a low energy (for MS scans) and an elevated energy (for fragmentation spectra, recorded without precursor selection). The spectral acquisition time at each energy setting was 0.5 s. The collision energy of the trap cell was alternated between 4 V (low energy MS scans) and 2 V (high energy fragmentation scans). In the low energy MS mode, the collision energy of the transfer cell was held constant at 4 V, while in the high-energy mode the applied collision energy was correlated with the mobility drift time of peptide precursors using the following values: for mobility bins 0–20, the collision energy was set to 17 V; for mobility bins 21–110, the collision energy was set in the range of 17–45 V; and for mobility bins 111–200, it ranged from 45–60 V. A solution of 0.2 µM Glu^1^-fibrinopeptide B (GFP) in 50% ACN with 0.1% FA was used as a lock-mass solution. The solution was delivered at a flow rate of 3 µL/min using an auxiliary pump of the LC system. The lock-mass data was sampled every 4 min using 0.5 sec scans over the same mass range.

**Data processing**. The LC/HDMS^E^ data were processed using PLGS (Protein Lynx Global Server) 3.0.2 (Waters Corp.). For each injection, all the HDMS^E^ data from each fractionation step were digitally combined into a single file using PLGS software. The low- and high-energy data were background subtracted, de-isotoped and charge-state reduced to the corresponding monoisotopic masses. Each monoisotopic mas was then lock-mass corrected to yield an accurate mass measurement. Fragment ions and their corresponding precursor ions were automatically aligned together based on their retention times and drift time profiles.^36^ Processed spectra were then searched against a custom protein database which was compiled from 16,648 Uniprot mouse protein sequences, the sequences of 4 spiked proteins (ADH, PHO, BSA, ENL), the sequence of porcine trypsin, and the heavy/light chain sequences of infliximab. The final custom database also included an equal number of entries of randomized sequences (one random sequence for each true sequence), resulting in a total of 33,314 entries in the database. The decoy strategy was used to control the false positive rate in HCP identification. Searches were limited to tryptic peptides with one potential missed cleavage, cysteine alkylation was considered a fixed modification, while methionine oxidation and N/Q deamidations were considered as variable modifications. The same mass tolerance of 20 ppm was allowed for the low–energy precursor ions as well as for the high-energy fragment ions.

## Supplementary Material

Supplemental_Figures.docx
