# Supplementary material for: Advanced assessment of the physicochemical characteristics of Remicade® and Inflectra® by sensitive LC/MS techniques
Source: MAbs. 2016 Jun 3;8(6):1021–34. doi: 10.1080/19420862.2016.1193661 (PMC4968138; doi:10.1080/19420862.2016.1193661)
Supplement: Supplemental_Figures.docx [file kmab-08-06-1193661-s001.docx]

Table S1. The oxidation levels of M255.

**Figure S1.** Amino acid sequence and peptic peptide coverage of infliximab. Each cyan bar under the sequence corresponds to a peptic peptide was identified and commonly found in all the four samples. A total of 100% linear coverage was obtained for infliximab. The coverage map was generated by DynamX 3.0 software.


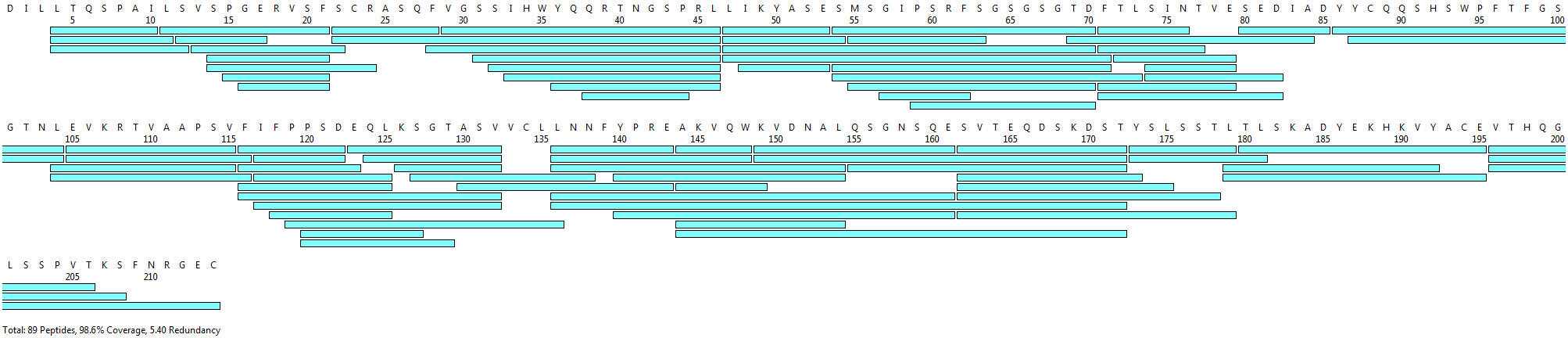


(A)

(B)


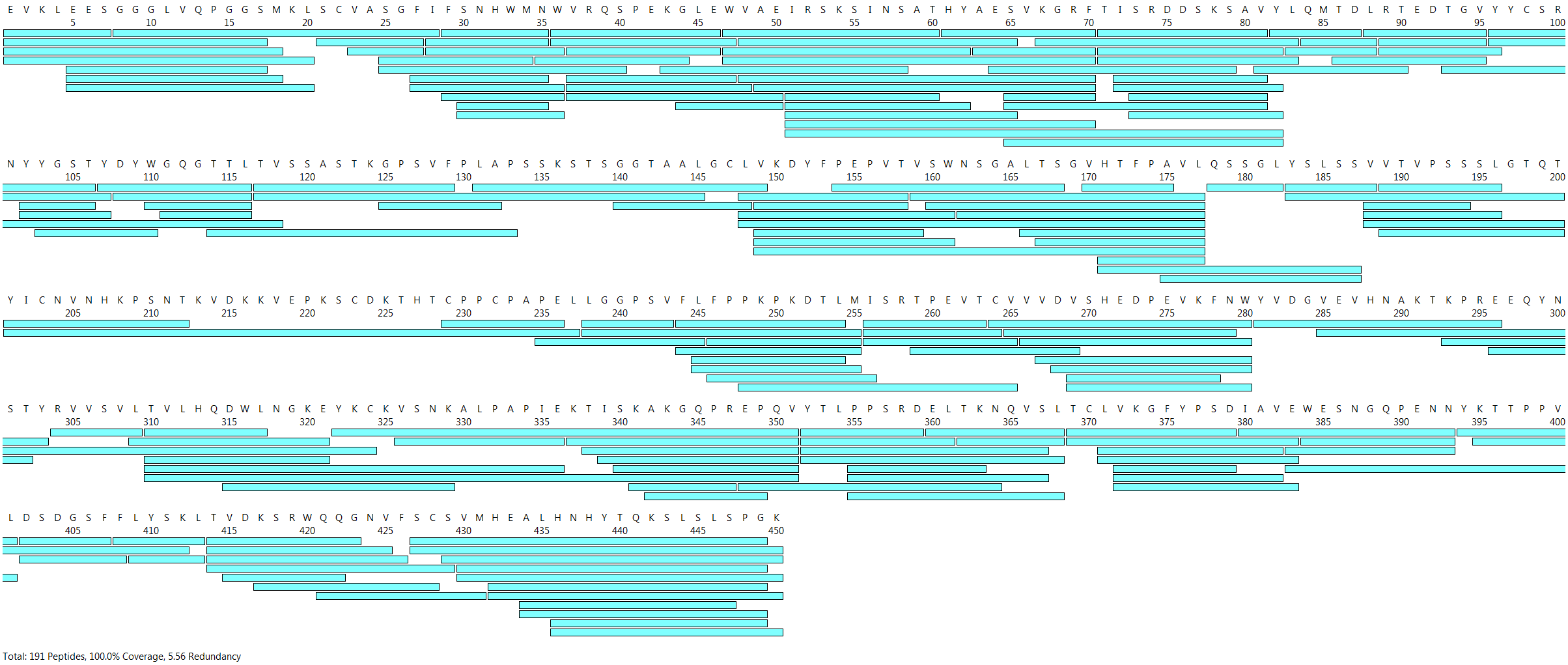

Figure S2. Deuterium incorporation information of innovator (A) and biosimilar (B) samples modeled to the structure of IgG1 (PDB 1HZH). The relative percent deuterium incorporation is shown at 30 s and 1, 10, 60, 240 min, respectively. The color code represents the relative fractional uptake.

Figure S3. BPI chromatograms of peptides generated by trypsin digestion of innovator lot 1 (top) and the biosimilar mAb (bottom).
